# Recombinant human follicle-stimulating hormone (r-hFSH) plus recombinant luteinizing hormone versus r-hFSH alone for ovarian stimulation during assisted reproductive technology: systematic review and meta-analysis

**DOI:** 10.1186/1477-7827-12-17

**Published:** 2014-02-20

**Authors:** Philippe Lehert, Efstratios M Kolibianakis, Christos A Venetis, Joan Schertz, Helen Saunders, Pablo Arriagada, Samuel Copt, Basil Tarlatzis

**Affiliations:** 1 Faculty of Economics, Université Catholique de Louvain (UCL Mons), 7000 Mons, Belgium; 2 Faculty of Medicine, the University of Melbourne, Melbourne 3010, Victoria, Australia; 3 Unit for Human Reproduction, 1st Department of Obstetrics and Gynaecology, Medical School, Aristotle University of Thessaloniki 54124 Thessaloniki, Greece; 4 Fertility Global Clinical Development Unit, EMD Serono, Inc, Rockland, MA 02370, USA (an affiliate of Merck KGaA, Darmstadt, Germany; 5 Formerly Merck Serono S.A, Geneva, Switzerland (an affiliate of Merck KGaA, Darmstadt, Germany; 6 Preglem SA, Chemin du Pré-Fleuri 3, 1228 Plan-les-Ouates, Geneva, Switzerland; 7 Biosensors, rue de Lausanne 31, 1100 Morges, Switzerland

**Keywords:** *In vitro* fertilization, Poor ovarian response, Pregnancy, Recombinant human follicle-stimulating hormone, Recombinant human luteinizing hormone supplementation

## Abstract

**Background:**

The potential benefit of adding recombinant human luteinizing hormone (r-hLH) to recombinant human follicle-stimulating hormone (r-hFSH) during ovarian stimulation is a subject of debate, although there is evidence that it may benefit certain subpopulations, e.g. poor responders.

**Methods:**

A systematic review and a meta-analysis were performed. Three databases (MEDLINE, Embase and CENTRAL) were searched (from 1990 to 2011). Prospective, parallel-, comparative-group randomized controlled trials (RCTs) in women aged 18–45 years undergoing *in vitro* fertilization, intracytoplasmic sperm injection or both, treated with gonadotrophin-releasing hormone analogues and r-hFSH plus r-hLH or r-hFSH alone were included. The co-primary endpoints were number of oocytes retrieved and clinical pregnancy rate. Analyses were conducted for the overall population and for prospectively identified patient subgroups, including patients with poor ovarian response (POR).

**Results:**

In total, 40 RCTs (6443 patients) were included in the analysis. Data on the number of oocytes retrieved were reported in 41 studies and imputed in two studies. Therefore, data were available from 43 studies (r-hFSH plus r-hLH, *n* = 3113; r-hFSH, *n* = 3228) in the intention-to-treat (ITT) population (all randomly allocated patients, including imputed data). Overall, no significant difference in the number of oocytes retrieved was found between the r-hFSH plus r-hLH and r-hFSH groups (weighted mean difference −0.03; 95% confidence interval [CI] −0.41 to 0.34). However, in poor responders, significantly more oocytes were retrieved with r-hFSH plus r-hLH versus r-hFSH alone (*n* = 1077; weighted mean difference +0.75 oocytes; 95% CI 0.14–1.36). Significantly higher clinical pregnancy rates were observed with r-hFSH plus r-hLH versus r-hFSH alone in the overall population analysed in this review (risk ratio [RR] 1.09; 95% CI 1.01–1.18) and in poor responders (*n* = 1179; RR 1.30; 95% CI 1.01–1.67; ITT population); the observed difference was more pronounced in poor responders.

**Conclusions:**

These data suggest that there is a relative increase in the clinical pregnancy rates of 9% in the overall population and 30% in poor responders. In conclusion, this meta-analysis suggests that the addition of r-hLH to r-hFSH may be beneficial for women with POR.

## Background

The efficacy of recombinant human follicle-stimulating hormone (r-hFSH) for ovarian stimulation is well established [1]; however, the role of supplementary recombinant human luteinizing hormone (r-hLH) is less clear. LH has a number of roles in follicular development [2] and in the periovulatory phase, LH is involved in the induction of ovulation [2], completion of meiosis I [3], early luteinization and the production of progesterone [4]. Ovarian steroidogenesis can be driven by activation of a low number (around 1%) of LH receptors and, during cycles of assisted reproductive technology (ART), adequate levels of endogenous LH are usually present despite pituitary suppression with gonadotrophin-releasing hormone (GnRH) analogues [5,6].

r-hLH in association with an FSH preparation is indicated for the stimulation of follicular development in adult women with severe LH and FSH deficiency [7]; in clinical trials these patients were defined by an endogenous serum LH level of <1.2 IU/l [8]. In addition to the utility of r-hLH supplementation in women with hypogonadotropic hypogonadism [9], evidence suggests that r-hLH supplementation may be beneficial for certain subpopulations of women; for example, those with an initial suboptimal (poor) ovarian response to r-hFSH monotherapy [9-11] and those aged >35 years [9,11]. Despite these potential benefits, the use of r-hLH supplementation during ovarian stimulation has long been debated and there is conflicting evidence in the literature [2].

The primary objective of the meta-analysis reported here was to compare the effectiveness of treatment with r-hFSH plus r-hLH with r-hFSH alone in infertile women undergoing ovarian stimulation with GnRH analogues. The investigation of the effect of r-hLH supplementation was also conducted in subpopulations of patients: for example, patients with a poor ovarian response (POR).

## Methods

The protocol used for this systematic review and meta-analysis (see Additional file 1: Supplementary Material A) adhered to the International Conference on Harmonisation (ICH) E9 Statistical Principles for Clinical Trials [12], the Cochrane Handbook for Systematic Reviews of Interventions [13] and the Committee for Proprietary Medicinal Products guidelines [14]. The project was initiated in November 2010 and completed in September 2011.

### Literature searches

Literature searches were conducted to identify studies published between 1 January 1990 and 1 May 2011. Three databases were searched: MEDLINE, Embase and CENTRAL. Google Scholar and relevant journals, symposia and conference proceedings were also used to identify further relevant publications. Non-published research (if available) could also be included as was any Merck Serono randomized controlled trial (RCT) known to be unpublished (prior to 2002). The search was not limited by language. The search strategy used key words/terms and database-specific indexing terminology (the MEDLINE search strategy is shown in Additional file 2: Table S1).

### Study selection

The inclusion criteria (established before the search) were: prospective, randomized, parallel-, comparative-group trials conducted in women aged 18–45 years undergoing *in vitro* fertilization (IVF), intracytoplasmic sperm injection (ICSI) or both, treated with GnRH analogues and r-hFSH plus r-hLH or r-hFSH alone for multifollicular development. Studies in patients or subgroups with anovulatory infertility or polycystic ovarian syndrome were excluded.

The titles of retrieved citations were initially reviewed by two authors to remove duplicates. The search results were cross-checked against publications listed in previous meta-analyses [15-19] to ensure that all relevant studies were included.

### Data collection

The eligibility and relevance of the trials were assessed by reviewing each abstract or the full text if the abstract was inadequate. If additional information was required, the corresponding authors and/or study sponsors were contacted.

To assess the methodological quality of RCTs, a qualitative checklist was completed and independently evaluated by each reviewer [13]. The checklist comprised seven items assessing internal, external and statistical validity (Additional file 1: Supplementary Material B).

The co-primary endpoints used for the meta-analysis were number of retrieved oocytes and clinical pregnancy rate, which was defined according to International Committee Monitoring Assisted Reproductive Technologies and the World Health Organization criteria as ultrasonographic visualization of one or more gestational sacs.

Other endpoints included: number of metaphase II oocytes, embryos and transferred embryos; positive β-human chorionic gonadotrophin test; ongoing pregnancy (defined as ultrasound evidence of at least one gestational sac with foetal cardiac activity); live birth (defined as the number of live births per started cycle); number of good quality embryos; duration of ovarian stimulation; peak oestradiol levels; and total dose of r-hFSH.

### Statistical methods

All statistical analyses were performed using R statistical packages (release 2.15.2).

The full analysis set from the studies was used because it is as close as possible to the intention-to-treat [ITT] principle of including all randomized patients. In this analysis, the ITT population consisted of all randomly allocated patients and included imputed data. In addition, the per-protocol (PP) population (patients from all studies in which the endpoint was fully documented) was used in supportive analyses.

The meta-analysis used a random effects model, which was calculated using both the restricted maximum likelihood (REML) and the DerSimonian and Laird approach [20]. Meta-regression on the ITT dataset considered pre-specified relevant covariates.

Four covariates were selected: 1) patient age – all patients (young/normal age, i.e. no selection regarding age) or advanced maternal age (>35 years); 2) ovarian response to treatment – normal or poor (POR); 3) mode of endogenous LH suppression – GnRH agonist or antagonist; and 4) insemination technique – IVF or ICSI. POR was defined according to study authors’ criteria and although the studies were published prior to European Society of Human Reproduction and Embryology (ESHRE) consensus definition for POR [21], in 10 of the 14 studies reporting POR data, the definition of POR employed was aligned with the subsequently reported ESHRE definition. Hierarchical clustering of studies was undertaken based on the first three covariates because most studies (*n* = 27; 60%) used both IVF and ICSI for the insemination technique.

For binary variables (e.g. clinical pregnancy), the risk ratio (RR) was evaluated as the main calculation of effect size [22]. Continuous variables (e.g. number of oocytes retrieved) were evaluated using the weighted mean difference, or the standardized mean difference (Cohen’s effect size) if the endpoints did not use the same measurement scale.

For missing endpoints, data were imputed using another endpoint related to the missing value as the covariate in a regression model to estimate the missing value. The calculations and coefficients for data imputation are shown in Additional file 3: Table S2. The linear relationship between the two variables was measured using the *R*^2^ and its 95% confidence interval (CI).

The internal and external validity of the meta-analysis were optimized by maximizing the sample size and controlling for bias. Sources of external bias were assessed to determine their possible impact on the observed effect size.

The risk of publication bias was assessed using the funnel plot method and analysed statistically using a linear regression test to determine the linear regression coefficient between log odds ratio (OR) and its standard error. Radial Galbraith plots were used to assess the consistency of the observed outcomes with different precisions (e.g. due to sampling variances).

See Additional file 1: Supplementary Material C for additional details of the statistical methods employed.

## Results

**Figure 1:**
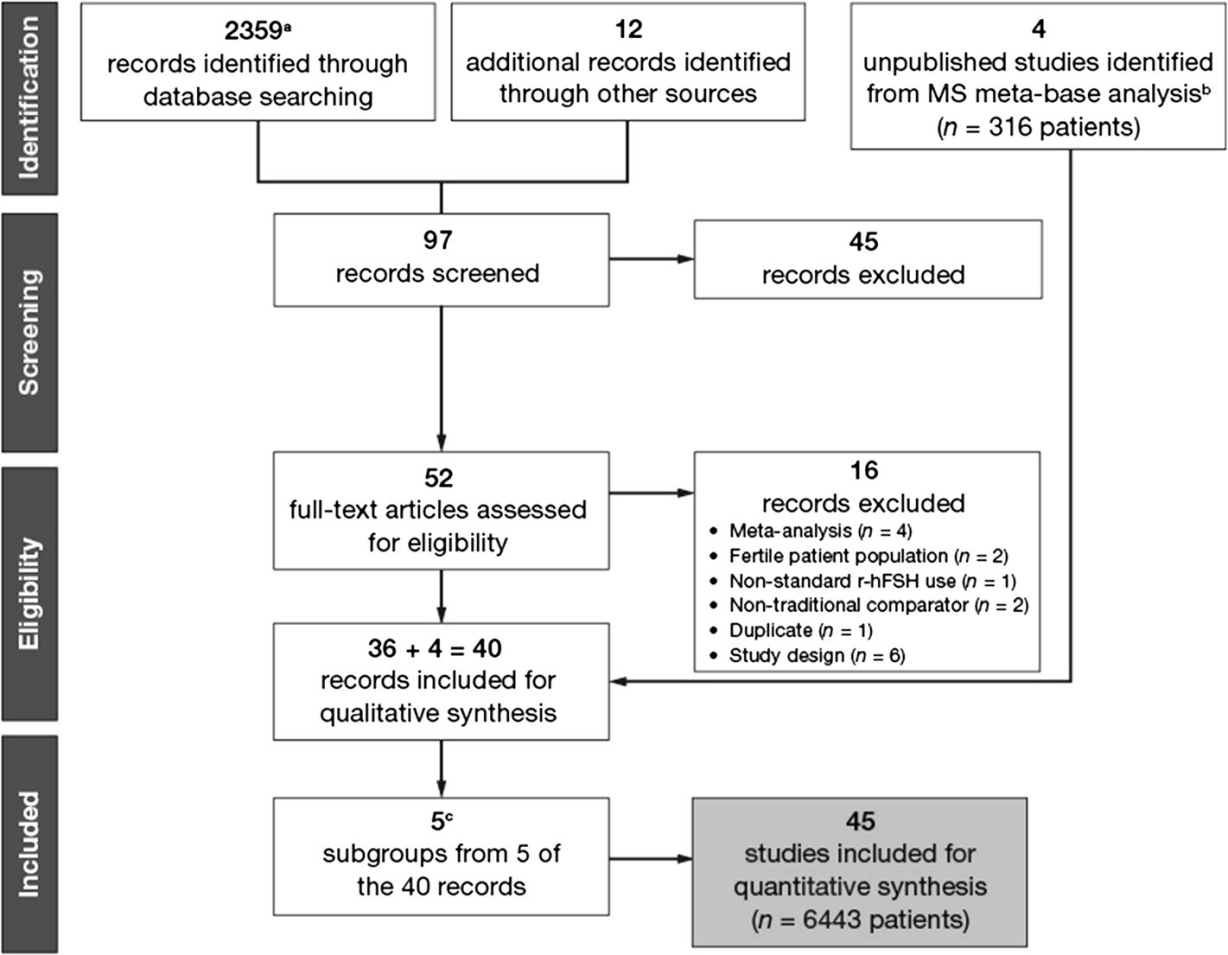
**The study selection process. **^a^2274 records excluded based on title. ^b^Studies MS8839, MS9029, MS9032 and MS9640. ^c^Age subgroups from Humaidan *et al*., 2004 [23]; Marrs *et al*., 2004 [25]; Nyboe Andersen *et al*., 2008 [28]; Bosch *et al*., 2011 [26]; gonadotrophin-releasing hormone analogue subgroup from Motta *et al*., 2005 [27]. MS, Merck Serono S.A. – Switzerland, an affiliate of Merck KGaA, Darmstadt, Germany; r-hFSH, recombinant human follicle-stimulating hormone.

Of the 2371 publications initially identified, 36 eligible published RCTs were included in the analysis (Figure 1). There were four relevant unpublished RCTs from the Merck Serono S.A. (Merck Serono S.A. – Switzerland, an affiliate of Merck KGaA, Darmstadt, Germany) database (study reference numbers: MS8839, MS9029, MS9032 and MS9640) and so these were also included. In total, data from 6443 patients undergoing ovarian stimulation for IVF/ICSI using r-hFSH plus r-hLH or r-hFSH alone (and a GnRH analogue) were available for analysis. Data for the co-primary endpoints were available for most (95.6%) studies. A summary of the studies (*n* = 40), including their subgroup categories, is shown in Table 1. Five RCTs included subgroups and these were considered as separate studies; thus, a total of 45 quantitative studies were included in the meta-analysis (Figure 1). In three studies [23-25], patients were divided according to young/normal versus advanced maternal age subgroups. In another study [26], the patient population in each group was prospectively stratified by age (young/normal versus advanced maternal age) and in another study [27], there were two subgroups classified according to the LH suppression method used (long GnRH agonist and GnRH antagonist protocol).

**Table 1:** **Main characteristics of the studies included in the meta**-**analysis** (***n*** = **40**)

**Study**	**Total number of patients in study**	**Patient response category**^**a**^	**Age category (age restrictions)**^**b**^	**GnRH agonist**, **GnRH antagonist or both**	**Insemination technique**	**Starting dose**		**Stimulation day (r-hLH initiated)**
						**r-hFSH (IU)**	**r-hLH (IU)**	
Williams 2000 [47]	60	Normal	None	Agonist	IVF/ICSI	300	25	
MS8839 [48]	76	Normal	None	Agonist	IVF	150	75	1
MS9032 [49]	45	Normal	None	Agonist	ICSI	^c^	75	1
MS9029 [50]	42	Poor	None	Agonist	ICSI	450	75	1
Balasch 2001 [51]	30	Normal	None	Agonist	IVF/ICSI	450	75	1
MS9640 [52]	141	Normal	None	Agonist	IVF/ICSI	225	75	1
Lisi 2002a [53]	453	Poor	None	Agonist	IVF	225	75	7
Lisi 2002b [54]	22	Normal	None	Agonist	IVF/ICSI	150	75	7
De Moustier 2002 [55]	169	Normal	Advanced	Agonist	IVF	225	^c^	
Ludwig 2003 [56]	20	Normal	None	Antagonist	IVF/ICSI		75	
Sauer 2004 [57]	42	Normal	None	Antagonist	ICSI	225	150	7
Cedrin-Durnerin 2004 [58]	203	Normal	None	Antagonist	IVF/ICSI	^c^	75	
Ferraretti 2004 [10]	108	Poor	None	Agonist	IVF/ICSI	^c^	^c^	
Humaidan 2004 [23]	231	Normal	Two subgroups	Agonist	IVF/ICSI	^c^	^c^	8
Marrs 2004 [25]	431	Normal	Two subgroups	Agonist	ICSI	225	150	6
Motta 2005 [27]	102	Poor	None	Both	IVF/ICSI		75	
Griesinger 2005 [59]	127	Normal	None	Antagonist	IVF/ICSI	150	75	1
Demirol 2005 [60]	106	Poor	None	Antagonist	ICSI	450	150	1
De Placido 2005 [61]	130	Poor	None	Agonist	IVF/ICSI	225	150	8
Tarlatzis 2006 [62]	114	Normal	None	Agonist	IVF/ICSI	150	75	
Ramirez 2006 [63]	34	Poor	None	Antagonist	IVF/ICSI	^c^	150	
Levi-Setti 2006 [64]	40	Normal	None	Antagonist	ICSI	225	75	
Abdelmassih 2006 [65]	206	Normal	None	Agonist	IVF/ICSI	225	75	7
Aytac 2006 [66]	35	Poor	None	Agonist	ICSI		150	
Fabregues 2006 [67]	120	Normal	Advanced	Agonist	IVF/ICSI	^c^	150	6
Ruvolo 2007 [68]	42	Poor	None	Agonist	IVF/ICSI	225	^c^	8
Polidoropoulos 2007 [69]	136	Poor	None	Agonist	ICSI	450	75	
Berkkanoglu 2007 [70]	97	Normal	None	Agonist	ICSI	600	75	7
Nyboe Andersen 2008 [24]	526	Normal	Two subgroups	Agonist	IVF/ICSI	^c^	^c^	7
Barrenetxea 2008 [71]	84	Poor	Advanced	Agonist	ICSI	375	150	6
Pezzuto 2010 [72]	80	Normal	None	Agonist	ICSI	^c^	75	6
Brunet 2009 [73]	94	Poor	None	Agonist	IVF		75	8
Gutman 2009 [74]	20	Normal	None	Agonist	IVF/ICSI	^c^	75	
Matorras 2009 [75]	131	Normal	Advanced	Agonist	ICSI	^c^	150	6
Lahoud 2010 [76]	103	Normal	None	Agonist	IVF/ICSI		75	7
Kovacs 2010 [77]	50	Normal	None	Agonist	IVF/ICSI	150	75	1
Wiser 2011 [78]	30	Normal	None	Antagonist	IVF/ICSI	^c^	75	
Musters 2012 [79]	244	Poor	Advanced	Agonist	IVF/ICSI	^c^	^c^	1
Caserta 2011 [80]	999	Normal	None	Agonist	ICSI	150	75	7
Bosch 2011 [26]	720	Normal	Two subgroups	Antagonist	IVF/ICSI	^c^	75	6
Total	6443							

^a^Normal = normal ovarian response, poor = poor/risk of poor ovarian response.

^b^Advanced = advanced maternal age, two subgroups = study had two age-based subgroups.

^c^Starting dose varied according to numerous factors, such as patient age or body mass index; blank spaces indicate that the data were not reported by the study author.

*GnRH*, gonadotrophin-releasing hormone; *ICSI*, intracytoplasmic sperm injection; *IVF*, *in vitro* fertilization; *r*-*hFSH*, recombinant human follicle-stimulating hormone; *r*-*hLH*, recombinant human luteinizing hormone.

Note: the study by Musters *et al*. was included in the analysis prior to its publication in 2012.

Nineteen studies reported their policy regarding the number of embryos that could be transferred: maximum of two embryos (*n* = 4); maximum of three embryos (*n* = 12); maximum of four embryos (*n* = 2); and in one study, the authors stated that they followed international guidelines although the maximum number of embryos transferred was not given.

No sources of publication bias were found. See Additional file 1: Supplementary Material D, Additional file 4: Figure S1 and Additional file 5: Figure S2 for the results of publication bias assessments and the consistency of the observed outcomes.

### Number of oocytes retrieved

Data on the number of oocytes retrieved were reported in 41 studies and imputed in two studies. Therefore, data were available from 43 studies (r-hFSH plus r-hLH, *n* = 3113; r-hFSH, *n* = 3228) in the ITT population (all randomly allocated patients, including imputed data). The PP population (the ‘available data’ subset) consisted of 41 studies (r-hFSH plus r-hLH, *n* = 3045; r-hFSH, *n* = 3194).

Overall, no significant difference in the number of oocytes retrieved was found between the r-hFSH plus r-hLH and r-hFSH groups in either the ITT population (mean difference: −0.03; 95% CI −0.41 to 0.34) or the PP population (mean difference: −0.03; 95% CI −0.40 to 0.34). Heterogeneity between studies was high (Q-test: *P* < 0.0001).

#### Covariate analyses

The patient’s ovarian response had a possible influence on the effect of r-hFSH plus r-hLH compared with r-hFSH alone for the number of oocytes retrieved, as a significant estimated effect on the number of oocytes retrieved was observed for r-hFSH plus r-hLH in poor (14 studies, *n* = 1179) versus normal (31 studies, *n* = 5264) responders: mean difference of 1.17 (*P* = 0.002; Table 2).

**Table 2:** **Results of meta**-**regression for the effect of subgroup and covariates analyses for number of oocytes and clinical pregnancy**

**Moderator (covariate)**^**a**^	**Number of oocytes**			**Clinical pregnancy**		
	**Difference**	**95% CI**	***P *** **value**	**RR**	**95% CI**	***P *** **value**
**Analysis of patient response subgroups**						
POR vs. normal responders	1.17	0.42 to 1.92	0.002	1.3	1.05 to 1.62	0.016
**Other analyses**						
Advanced maternal age (>35 years) vs. younger age	−0.66	−1.51 to 0.20	0.132	1.1	0.90 to 1.33	0.378
Typology (NPG class vs. others^b^)	1.40	0.35 to 2.46	0.009	1.3	0.98 to 1.75	0.067
Missing data: imputed vs existing data	1.52	−3.68 to 6.73	0.566	1.3	0.76 to 2.23	0.332
Publication year	−0.04	−0.16 to 0.09	0.577	1.0	0.97 to 1.03	0.934
Published vs. unpublished (congress abstracts)	0.87	−0.76 to 2.51	0.296	0.7	0.44 to 1.25	0.262
Published vs. unpublished (full papers)	0.27	−1.22 to 1.75	0.724	0.8	0.48 to 1.30	0.346
Methodological quality score (MQS)	−0.96	−2.44 to 0.52	0.204	0.9	0.68 to 1.30	0.693
Sponsored vs. non-sponsored studies	−0.35	−1.15 to 0.45	0.394	0.9	0.78 to 1.09	0.346
Multicentre vs. single centre	0.36	−0.45 to 1.17	0.386	0.9	0.80 to 1.12	0.517
ART technique (ICSI vs. IVF)	0.21	−0.17 to 0.59	0.281	1.1	0.94 to 1.33	0.212
GnRH antagonist vs. GnRH agonist	−0.12	−1.02 to 0.77	0.787	0.9	0.78 to 1.10	0.364

^a^With the exception of continuous variables, the categories of the meta-regressors were binary, e.g. POR vs. normal responders.

^b^Other typology groups: NNG (young/normal age, normal response, GnRH agonist); ANG (advanced maternal age, normal response, GnRH agonist); NNN (young/normal age, normal response, GnRH antagonist); NPN (young/normal age, poor response, GnRH antagonist); APG (advanced maternal age, poor response, GnRH agonist); and ANN (advanced maternal age, normal response, GnRH antagonist).

*ART*, assisted reproductive technology; *CI*, confidence interval; *GnRH*, gonadotrophin-releasing hormone; *ICSI*, intracytoplasmic sperm injection; *IVF*, *in vitro* fertilization; NPG, young/normal age, poor response, GnRH agonist; *POR*, poor ovarian response; *RR*, risk ratio.

The results of other covariate analyses for number of oocytes retrieved are shown in Table 2.

#### Subgroup analyses

**Figure 2:**
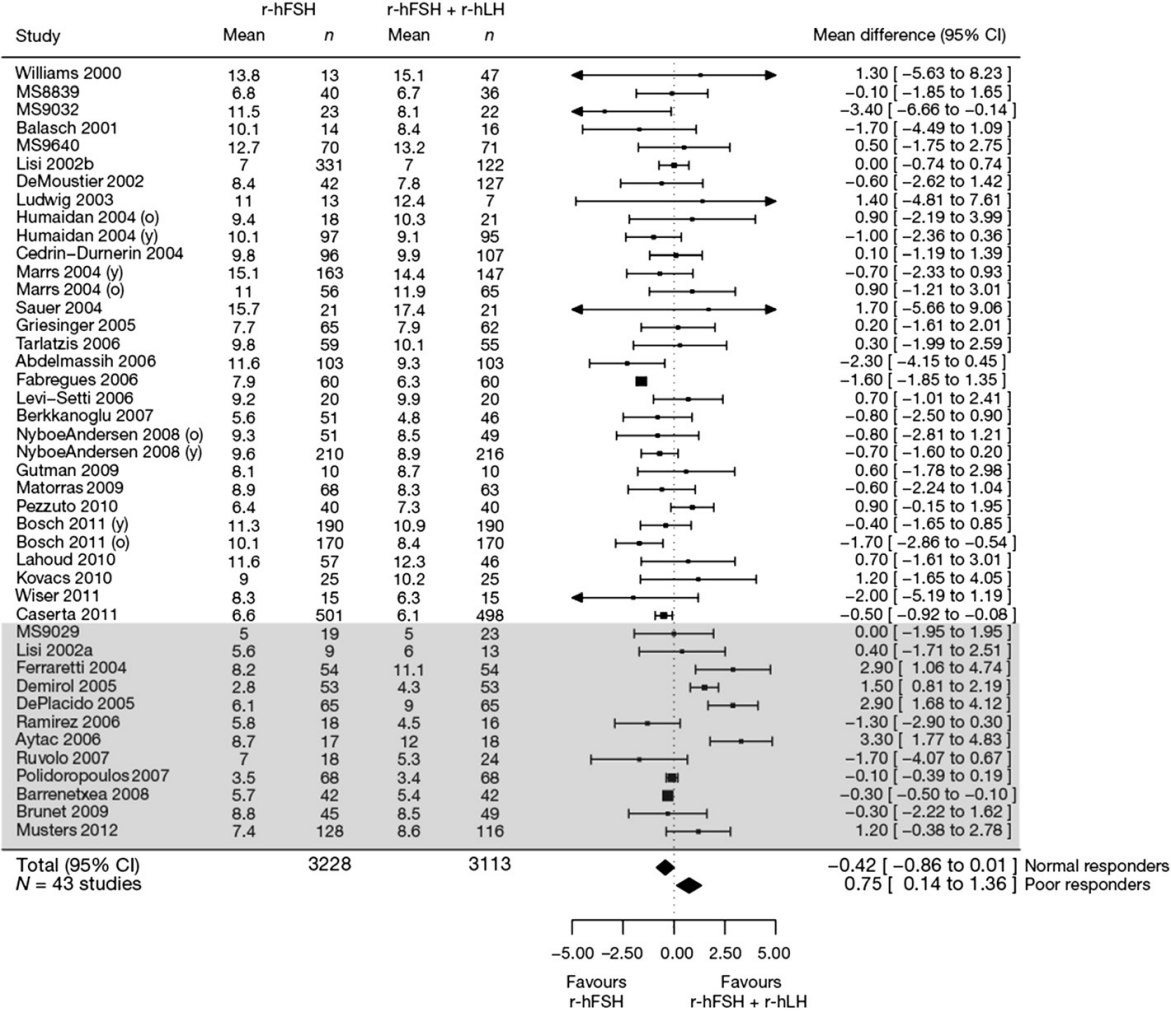
**Forest plot of the number of oocytes retrieved in normal versus poor responders (intention-to-treat population).** Studies are listed by first author’s last name followed by the year of publication. Some studies were divided by subgroup designations: y, young/normal or o, advanced maternal age. The grey-shaded box designates studies of patients with a poor ovarian response. CI, confidence interval; MS, Merck Serono S.A. – Switzerland, an affiliate of Merck KGaA, Darmstadt, Germany; r-hFSH, recombinant human follicle-stimulating hormone; r-hLH, recombinant human luteinizing hormone.

The results of the subgroup analysis for normal and poor responders are shown in Figure 2. In the ITT population, a significant benefit on the number of oocytes retrieved was found for r-hFSH plus r-hLH versus r-hFSH alone in poor responders (12 studies, *n* = 1077 [data for the two studies conducted by Motta *et al*., 2005 [27] could not be imputed]); mean difference +0.75 oocytes (95% CI 0.14–1.36). The results in the PP population were consistent with those of the ITT population, with a significant benefit of +0.75 oocytes (95% CI 0.13–1.36) for r-hFSH plus r-hLH versus r-hFSH alone in poor responders.

A non-significant negative effect was observed for r-hFSH plus r-hLH versus r-hFSH alone in normal responders (31 studies, *n* = 5264): mean difference −0.42 oocytes (95% CI −0.86 to 0.01) in the ITT population and −0.44 oocytes (95% CI −0.87 to 0.00) in the PP population.

Study typology analysis of r-hFSH plus r-hLH versus r-hFSH alone for the number of oocytes retrieved (Additional file 6: Table S3) found a significant benefit for the subgroup of patients who were young/normal age, with a poor response, and received GnRH agonist (mean difference +1.40 oocytes; 95% CI 0.35–2.46; *P* = 0.01).

### Clinical pregnancy rate

Data on clinical pregnancy rate were reported for 39 studies and imputed for four studies; therefore, data were available from 43 studies (r-hFSH plus r-hLH, *n* = 3139; r-hFSH, *n* = 3254) in the ITT population and 39 studies (r-hFSH plus r-hLH, *n* = 3065; r-hFSH, *n* = 3172) in the PP population.

A significant benefit of r-hFSH plus r-hLH over r-hFSH alone was found for clinical pregnancy rate: RR 1.09 (95% CI 1.01–1.18) in the overall ITT population. The RR for this variable for r-hFSH plus r-hLH versus r-hFSH alone in the PP population was not significant (1.09 [95% CI 1.00–1.19]).

Heterogeneity between studies was low for RR (Q-test: *P* = 0.437; *I*^2^ [percentage of total variability due to heterogeneity] 1.85%).

#### Covariate analyses

There was a significant increase in clinical pregnancy rate (RR 1.3; 95% CI 1.05–1.62; *P* = 0.016) with r-hFSH plus r-hLH versus r-hFSH alone in poor responders compared with normal responders (Table 2).

The results of the other covariate analyses for clinical pregnancy rate are given in Table 2.

#### Subgroup analyses

**Figure 3:**
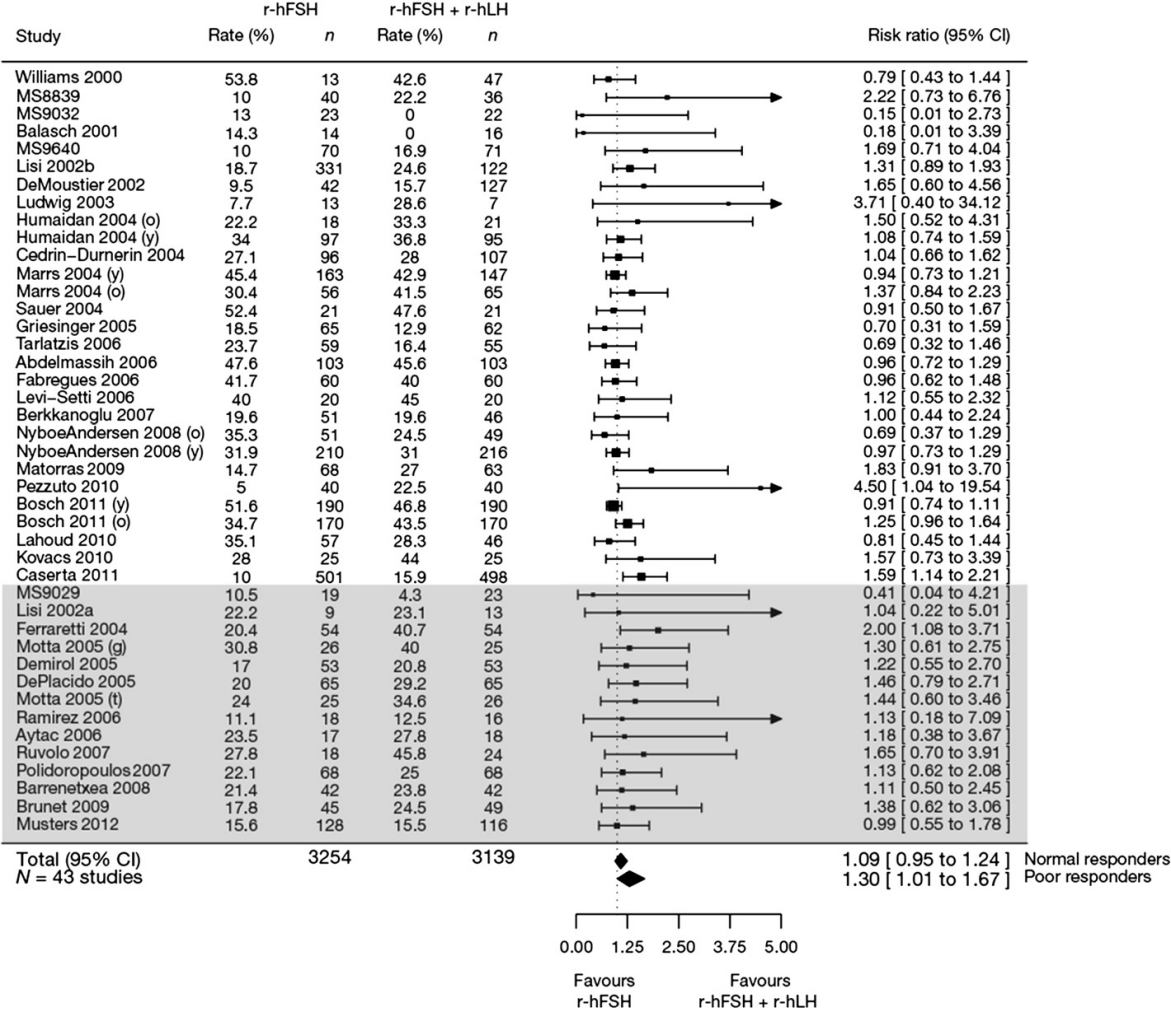
**Forest plot of the clinical pregnancy rate for normal versus poor responders (intention-to-treat population).** Studies are listed by first author’s last name followed by the year of publication. Some studies were divided by subgroup designations: y, young/normal or o, advanced maternal age; g, GnRH agonist or t, GnRH antagonist. The grey-shaded box designates studies of patients with a poor ovarian response. CI, confidence interval; MS, Merck Serono S.A. – Switzerland, an affiliate of Merck KGaA, Darmstadt, Germany; r-hFSH, recombinant human follicle-stimulating hormone; r-hLH, recombinant human luteinizing hormone.

A significant benefit on the clinical pregnancy rate was found for r-hFSH plus r-hLH versus r-hFSH alone in poor responders (14 studies, *n* = 1179): RR 1.30 (95% CI 1.01–1.67) in the ITT population (Figure 3). In the PP population, the results were not significant: RR 1.29 (95% CI 0.96–1.73).

A non-significant difference in clinical pregnancy rates for r-hFSH plus r-hLH versus r-hFSH alone was observed in normal responders (29 studies): RR 1.09 (95% CI 0.95–1.24). Similar results were obtained in the PP population (28 studies): RR 1.09 (95% CI 0.94–1.26).

Study typology analysis found no significant benefits on clinical pregnancy rate for either r-hFSH plus r-hLH or r-hFSH (Additional file 6: Table S3).

### Secondary endpoints

#### Ongoing pregnancy rate

Ongoing pregnancy rate was reported in 14/45 studies and data imputation was performed for 25 other studies; there were 3065 and 3172 patients in the r-hFSH plus r-hLH and r-hFSH groups, respectively (ITT population). The gestational age used for ongoing pregnancy was reported in nine studies and ranged from 10 to 20 weeks. The RR for ongoing pregnancy rate was significant in favour of r-hFSH plus r-hLH (1.14; 95% CI 1.05–1.25; Table 3). In the PP population, the RR for ongoing pregnancy rate was not significant.

**Table 3:** **Outcomes of other efficacy endpoints investigated in the meta**-**analysis in the overall population and in the normal and poor response subgroups** (**difference between r**-**hFSH plus r**-**hLH and r**-**hFSH alone groups**; **ITT population**)

	**Mean difference/RR (95% CI) between r-hFSH plus r-hLH and r-hFSH groups**		
	**Overall ITT population**	**Poor responders**	**Normal responders**
Number of metaphase II oocytes	0.02 (−0.29 to 0.33)^a^	**0.69 (0.12** to **1.25)**^a^	−0.28 (−0.66 to 0.10)^a^
Number of embryos	0.09 (−0.11 to 0.30)^a^	0.34 (−0.05 to 0.73)^a^	−0.01 (−0.27 to 0.25)^a^
Number of transferred embryos	**0.09 (0.01** to **0.17)**^a^	**0.27 (0.07** to **0.47)**^a^	0.05 (−0.05 to 0.15)^a^
Number of good quality embryos	**0.26 (0.16** to **0.36)**^a^	**0.43 (0.26** to **0.06)**^a^	**0.17 (0.05** to **0.29)**^a^
Peak oestradiol (ng/L)	**0.24 (0.06** to **0.42)**^a^	0.21 (−0.12 to 0.54)^a^	**0.26 (0.03** to **0.48)**^a^
Duration of ovarian stimulation (days)	−0.23 (−0.50 to 0.05)^a^	−0.51 (−1.15 to 0.12)^a^	−0.15 (−0.49 to 0.18)^a^
Total r-hFSH dose (IU/1000)	−0.11 (−0.22 to 0.00)^a^	−0.38 (−0.59 to −0.17)^a^	−0.06 (−0.16 to 0.04)^a^
Biochemical pregnancy rate	**1.25 (1.13** to **1.38)**^b^	**1.38 (1.06** to **1.80)**^b^	**1.22 (1.04** to **1.42)**^b^
Ongoing pregnancy rate	**1.14 (1.05** to **1.25)**^b^	**1.36 (1.04** to **1.79)**^b^	1.13 (1.00 to 1.27)^b^
Live birth rate	**1.11 (1.01** to **1.21)**^b^	1.30 (0.95 to 1.78)^b^	1.10 (0.94 to 1.29)^b^

^a^Mean difference (95% CI).

^b^RR (95% CI).

*CI*, confidence interval; *ITT*, intention-to-treat; *r*-*hFSH*, recombinant human follicle-stimulating hormone; *r*-*hLH*, recombinant human luteinizing hormone; *RR*, risk ratio.

Note: bold denotes statistical significance for r-hFSH plus r-hLH versus r-hFSH alone.

In poor responders (11 studies; 1043 patients), a statistically significant benefit was observed for r-hFSH plus r-hLH for ongoing pregnancy rate (RR 1.36; 95% CI 1.04–1.79; Table 3). A non-significant benefit for r-hFSH plus r-hLH for ongoing pregnancy rate was observed in normal responders (RR 1.13; 95% CI 1.00–1.27; Table 3).

#### Live birth rate

Live birth rates were reported for 8/45 studies and data were imputed for 31 studies; *n* = 3065 and *n* = 3172 patients in the r-hFSH plus r-hLH and r-hFSH groups, respectively (ITT population). The RR for live birth rate was statistically significant in favour of r-hFSH plus r-hLH (1.11 [95% CI 1.01–1.21]; Table 3). In the PP population, there was a non-significant benefit in favour of r-hFSH plus r-hLH for live birth rate.

A non-significant benefit for r-hFSH plus r-hLH on live birth rate was observed in both poor (RR 1.30; 95% CI 0.95–1.78) and normal (RR 1.10; 95% CI 0.94–1.29) responders (Table 3).

#### Other efficacy endpoints

The outcomes of other efficacy endpoints in the r-hFSH plus r-hLH and r-hFSH treatment groups in the ITT population are shown in Table 3.

## Discussion

To date, this meta-analysis is the most comprehensive compilation of data to assess the outcomes of r-hFSH plus r-hLH or r-hFSH alone for ovarian stimulation during ART. Our findings indicate that there was no significant difference in the number of oocytes retrieved with r-hFSH plus r-hLH versus r-hFSH alone in the overall population studied. However, we also found that significantly more oocytes were retrieved in women treated with r-hFSH plus r-hLH versus r-hFSH alone in the subgroup of poor responders (+0.75; 95% CI 0.14–1.36). In addition, a significant benefit of r-hFSH plus r-hLH versus r-hFSH alone on clinical pregnancy rate was demonstrated in the poor responders subgroup (RR 1.30; 95% CI 1.01–1.67), which suggests a 30% relative increase in clinical pregnancy rate among poor responders who received r-hLH supplementation. In the overall pooled population analysed here, a significant difference in clinical pregnancy rate was also found in favour of r-hFSH plus r-hLH versus r-hFSH alone (RR 1.09; 95% CI 1.01–1.18). This suggests that there was a smaller (estimate of 9%) relative increase in clinical pregnancy rate among all patients who received r-hLH supplementation compared with that seen in poor responders.

Our findings in the subgroup of poor responders are particularly relevant because many patients undergoing ART are poor responders to ovarian stimulation (although prevalence estimates vary because of differences in the definitions of POR used). Ferraretti and colleagues reported that approximately 33% of patients aged <30–39 years undergoing ovarian stimulation were poor responders (patients with <4 oocytes retrieved) [21]. In addition, evidence in the literature to identify interventions that could improve treatment outcomes in women with POR is limited [28-30] and the traditional clinical approach of increasing the FSH dose to improve follicular response appears to be ineffective [31-33]. Some physicians have attempted to exploit the potential benefit of r-hLH supplementation in women with POR. However, currently, women with POR may undergo multiple unsuccessful ART treatment cycles because of inadequate follicular response, repeated cycle cancellation or a negative pregnancy test.

The use of r-hLH supplementation during ovarian stimulation is a subject of debate in the literature and this lack of clarity has led to the publication of a number of earlier meta-analyses. The first showed a beneficial effect of ‘LH activity’ versus r-hFSH on clinical pregnancy rates (RR 1.18; 95% CI 1.02–1.36) in normogonadotropic women who underwent GnRH agonist downregulation [34]. In contrast to those results and the findings of our meta-analysis, three subsequent meta-analyses reported no clinical benefit of LH supplementation: no statistically significant differences were observed with r-hFSH plus r-hLH versus r-hFSH alone in pregnancy [15], live birth [16] or clinical pregnancy [18] rates. Another meta-analysis conducted in women of advanced reproductive age (≥35 years) found that the clinical pregnancy rate was higher in the r-hLH supplementation group than in the r-hFSH alone group (seven studies, *n* = 902; OR 1.37; 95% CI 1.03–1.83) [35]. In agreement with the findings of our meta-analysis, an additional meta-analysis found a statistically significant higher pregnancy rate in favour of r-hFSH plus r-hLH compared with r-hFSH alone in a subgroup of patients with poor ovarian response (POR) (three studies; *n* = 310; OR 1.85; 95% CI 1.10–3.11) [17]. It should be noted that until the development of the ESHRE consensus POR definition in 2011 [21], heterogeneous definitions of POR were used.

In the current meta-analysis, the chosen co-primary endpoints were number of retrieved oocytes and clinical pregnancy. Of the 45 studies analysed, 41 reported data for number of oocytes, and 39 reported data for clinical pregnancy. Although live birth rate is the outcome measure that patients are most interested in, data for this endpoint were reported sporadically in the RCTs. Oocytes are the direct physiological result of ovarian stimulation by FSH and so reflect the pharmacological effect of FSH, therefore, the number of oocytes retrieved is an appropriate endpoint. Furthermore, the number of oocytes retrieved is not influenced by events occurring after oocyte retrieval, such as fertilization, implantation, or embryo/foetal development, whereas other endpoints of cycle success, such as pregnancy outcomes and live birth rates, may be impacted by these other events.

In addition, number of oocytes retrieved has been widely used as a measure of ovarian response to FSH stimulation and is commonly referenced as a predictor of successful ART outcomes. Increase in pregnancy rates associated with an increased number of oocytes retrieved has been reported by numerous authors through large and smaller retrospective analyses of IVF, ICSI and oocyte donation cycles [36-43]. Sunkara and co-workers (using data from 400,135 ART cycles) found a strong association between live birth rate and number of oocytes retrieved, with live birth rate increasing as the number of oocytes retrieved increased (up to 15 oocytes retrieved) [41]. For patients with a low number (three or fewer) of oocytes retrieved, an increase in live birth rate was observed when as few as one additional oocyte was retrieved [41]. Also, for patients aged ≥40 years with a low number of oocytes retrieved, an increase of just one oocyte had a marked increase in the predicted live birth rate. In addition, an evaluation of nearly 8000 ART cycles found the ongoing pregnancy rate to be highly correlated with the number of oocytes retrieved [44]. These findings suggest that in patients who may be expected to have lower numbers of oocytes retrieved, for example patients with POR, an increment of one additional oocyte might have a significant effect on pregnancy outcomes, such as ongoing pregnancy rate and live birth rate. Supporting this, a recent systematic review reported that the likelihood of pregnancy is reduced in women with POR when fewer oocytes are retrieved (pregnancy rate per started cycle of 0–7% with one oocyte, compared with 11.5–18.6% with four oocytes) [45].

The meta-analysis reported here found an increase in clinical pregnancy rate with r-hLH supplementation in the overall study population of women undergoing ovarian stimulation, and this finding has been reported in one previous meta-analysis [34]. However, this was not observed in two other meta-analyses [15,18] that involved fewer studies and smaller numbers of patients than the analysis reported here.

Our analysis attempted to utilize all available data, by imputing missing secondary endpoints, so that the sample size for each endpoint was the best possible for all endpoints. Data imputation for uncommon endpoints, such as live birth rate, may be viewed with caution by some, however, we felt that this practice was justified to allow a greater sample size to be analysed for live birth rate. In contrast to the statistically significant difference in clinical pregnancy rates between r-hFSH plus r-hLH versus r-hFSH alone in poor responders in the ITT population, the difference between the two treatments was not significant in poor responders in the PP population, although the effect size was similar. Data were imputed for the ITT population but not for the PP population.

It is interesting to note that a non-significant negative bias was identified for trials sponsored by the pharmaceutical industry, with a smaller effect of r-hFSH plus r-hLH versus r-hFSH alone in industry-sponsored trials compared with non-sponsored trials. This contrasts with previous findings of a significant positive outcome bias related to industry sponsorship [46]. In addition, the decision to include unpublished data was made to enable evaluation of as much data as possible in the analysis to provide a complete picture of the use of r-hFSH plus r-hLH compared with r-hFSH alone. Furthermore, we conducted analyses of various covariates, including ones for publication status (unpublished data versus congress abstract and unpublished data versus peer-reviewed paper).

The key strength of this meta-analysis is that it comprised the largest number of studies (to the best of our knowledge, all studies) on this subject. In addition, no *a priori* selection was admitted and bias control was systematically conducted through meta-regression.

A possible limitation of the current meta-analysis is that the 14 studies of women with POR that were included had been conducted prior to the publication of the ESHRE consensus POR definition in 2011 [21]. Accordingly, heterogeneous definitions of POR were used in these studies. When comparing the study authors’ definitions of POR with the ESHRE consensus criteria [21], each of the studies in the POR analysis were aligned with at least one of the ESHRE criteria and the ESHRE definition of POR was reflected (through alignment with at least two ESHRE criteria) in 10 of these studies.

## Conclusions

This systematic review and meta-analysis suggests that r-hLH supplementation of r-hFSH compared with r-hFSH alone may result in benefits in terms of clinical pregnancy rate in the overall pooled population, as well as in poor responders. In addition, a benefit for r-hFSH plus r-hLH versus r-hFSH alone may be seen for the number of oocytes retrieved in poor responders.

## Abbreviations

ART: Assisted reproductive technology; CI: Confidence interval; ESHRE: European Society of Human Reproduction and Embryology; FSH: Follicle-stimulating hormone; GnRH: Gonadotrophin-releasing hormone; ICH: International Conference on Harmonisation; ICSI: Intracytoplasmic sperm injection; ITT: Intention-to-treat; IVF: *In vitro* fertilization; LH: Luteinizing hormone; OR: Odds ratio; POR: Poor ovarian response; PP: Per-protocol; RCT: Randomized controlled trial; REML: Restricted maximum likelihood; r-hLH: recombinant human luteinizing hormone; r-hFSH: recombinant human follicle-stimulating hormone; RR: Risk ratio.

## Competing interests

PL has received fees from Merck KGaA for conducting this analysis. BT has the following potential conflicts of interest: consultancy, lectures/speakers bureau – Ferring, Institut Biochimique SA (IBSA) and Merck Sharp & Dohme; consulting fee/honorarium, review activities, consultancy, lectures/speakers bureau – Merck Serono. EMK has the following potential conflicts of interest: lectures/speakers bureau, travel/accommodations/meeting expenses – Ferring; consulting fee/honorarium – Merck Serono and Merck Sharp & Dohme. CAV has the following potential conflicts of interest: travel/accommodations/meeting expenses – Ferring; consultancy, lectures/speakers bureau, development of educational presentations – Ipsen; consulting fee/honorarium, review activities – Merck Serono; travel/accommodations/meeting expenses – Merck Sharp & Dohme. PA, SC and HS were employees of Merck Serono S.A. – Switzerland, an affiliate of Merck KGaA, Darmstadt, Germany, at the time of the study. JS is an employee of EMD Serono, Inc., Rockland, MA, USA (an affiliate of Merck KGaA, Darmstadt, Germany).

## Authors’ contributions

All authors contributed to manuscript drafting and critical discussion of the manuscript. JS and HS performed the literature search and initial review of titles of retrieved citations as well as the initial assessment of the eligibility and relevance of trials by reviewing each abstract. If study eligibility was unclear, PL, BT, EMK, CAV, JS, HS and SC inspected full text versions and any disagreement was resolved through discussion. Data extraction was performed independently by BT, EMK, CAV, JS and HS. PL, BT, EMK, CAV, JS, HS and SC completed and independently evaluated a qualitative checklist (see Additional file 7, PRISMA checklist) of questions relating to the internal, external and statistical characteristics of each trial. PA provided expert medical advice, contributed to the study methodology and planning, and reviewed the clinical variables and publications before and after data extraction. PL and SC contributed to the statistical methodology and PL performed all data analyses. All authors read and approved the final manuscript.

## Supplementary Material

Additional file 1 Supplementary Material A-D. Click here for file

Additional file 2: Table S1 Search strategy for the MEDLINE database. Click here for file

Additional file 3: Table S2 Missing data imputation calculations and coefficient determinations. Click here for file

Additional file 4: Figure S1 Funnel plot of effect size by standard error for number of oocytes. Click here for file

Additional file 5: Figure S2 Radial Galbraith plot for number of oocytes to assess the consistency of the observed outcomes with different precisions. Click here for file

Additional file 6: Table S3 Study typology analysis for the co-primary endpoints (difference between the r-hFSH plus r-hLH and r-hFSH alone groups). Click here for file

Additional file 7 PRISMA checklist. Click here for file
