# Supplementary material for: Recombinant human follicle-stimulating hormone (r-hFSH) plus recombinant luteinizing hormone versus r-hFSH alone for ovarian stimulation during assisted reproductive technology: systematic review and meta-analysis
Source: Reprod Biol Endocrinol. 2014 Feb 20;12:17. doi: 10.1186/1477-7827-12-17 (PMC4015269; doi:10.1186/1477-7827-12-17)
Supplement: Additional file 1 — Supplementary Material A-D. [file 1477-7827-12-17-S1.docx]

**Supplementary Material**

**A. Protocol**

**B. Methodological quality checklist**

**Internal validity**

Was the assigned treatment adequately concealed prior to allocation?

Were the outcomes of patients who withdrew or were excluded after allocation described and included in an ‘intention-to-treat’ analysis?

Were the outcome assessors blind to assignment status?

Were the participants blind to assignment status following allocation?

Were the treatment providers blind to assignment status?

Were the care programs, other than the trial options, identical?

Were the withdrawals <10% of the trial population?

**External validity**

Were the inclusion and exclusion criteria for entry clearly defined?

Were the outcome measures used clearly defined?

Were the accuracy, precision and observer variation of the outcome measures adequate?

Was the timing of the outcome measures appropriate?

Was the quality of allocation concealment graded?

**Statistical validity**

Was the main endpoint mentioned?

Was the power analysis described?

Was the baseline comparison reported?

Was the statistical technique appropriate?

Were the table and graphical results clear?

**Supplementary Material**

**C. Additional statistical methods**

The fixed effects model was used only for sensitivity purposes and when non-significant heterogeneity between studies was found (Q-test, *P* > 0.05 and *I*^2^ index <0.25). The pooling principle was invariably based on the mean difference weighted by the inverse of its variance.

*Subgroups and covariates*: Four covariates were selected: 1) patient age – all patients (young/normal age, i.e. no selection regarding age) or advanced maternal age (>35 years); 2) ovarian response to treatment – normal or poor (POR was defined according to study authors’ criteria); 3) mode of endogenous LH suppression – GnRH agonist or antagonist; and 4) insemination technique – IVF or ICSI. Hierarchical clustering of studies was undertaken based on the first three covariates. Insemination technique was not included because most studies (*n =* 27; 60%) used both IVF and ICSI. Under cluster analysis, studies were classified into seven trial typology groups to compare the relative effect of r-hFSH plus r-hLH versus r-hFSH alone: 1) NNG (young/normal age, normal response, GnRH agonist), considered to be the control group; 2) NPG (young/normal age, poor response, GnRH agonist); 3) ANG (advanced maternal age, normal response, GnRH agonist); 4) NNN (young/normal age, normal response, GnRH antagonist); 5) NPN (young/normal age, poor response, GnRH antagonist); 6) APG (advanced maternal age, poor response, GnRH agonist); and 7) ANN (advanced maternal age, normal response, GnRH antagonist). No eligible studies were performed in women with advanced age who were poor responders using GnRH antagonist protocols.

In addition, the groups were analysed in terms of poor versus normal ovarian response (NPG + NPN + APG versus NNG + NNN + ANG + ANN), normal versus advanced maternal age (NNG + NPN + NNN versus ANG + ANN + APG), and GnRH antagonist versus agonist (NNN + NPN + ANN versus NNG + NPG + ANG + APG).

#### Co-primary endpoints

The multiplicity of type I error and the further decision to use a double endpoint was managed through a step-down approach. This required a significant superiority of r-hFSH plus r-hLH versus r-hFSH on the number of oocytes before questioning the superiority of r-hFSH plus r-hLH on the clinical pregnancy rate (because of the putative proposition that the number of oocytes is the essential mediator of clinical pregnancy rate). Under these conditions, the two subsequent tests of superiority were tested using the same alpha level (two-sided, *P* = 0.05) and the overall experiment-wise type I error was bounded by this level [1]. In addition to this step-down procedure, the conservative Bonferroni rule was used in requesting a two-side significance of at least *P* = 0.025 for both endpoints.

#### Sources of bias

The methodological quality of the studies was assessed using a domain-based evaluation (DBE) of three domains: internal, external, and statistical validity (see above). Sensitivity analyses were conducted on the basis of the methodological quality. Thus, in addition to DBE, a methodological quality score (MQS) was created. A principal components analysis was conducted on the three domains. As the first component explained 84% of the whole variance, a one-dimensional sum-score index of MQS was used throughout the analysis [2].

In addition, other possible confounders were assessed: missing data (where appropriate), publication year, industry sponsorship, publication status (unpublished data versus congress abstract and versus peer-reviewed paper) and single- or multicentre trial design. For every endpoint, the significance of each of these effects and their direction was assessed before the analysis was conducted.

**D. Publication bias and consistency of observed outcomes**.

*Number of oocytes:* No sources of publication bias were identified. There was no apparent asymmetry in the configuration of points with regard to the vertical axis of null difference in a funnel plot of effect size and standard error (Supplementary Figure 1); although three studies were characterized by a small standard error, this did not result in a statistically significant effect. A radial Galbraith plot (Supplementary Figure 2), to assess the consistency of the observed outcomes with different precisions, showed that most points remained within the tolerance zone and, thus, did not provide suspicion of bias in a particular direction due to different precisions. No sources of external bias were identified (Table 2).

*Clinical pregnancy rate*: No sources of publication bias were identified. There was no apparent asymmetry in the configuration of points with regard to the vertical axis of null difference in a funnel plot. A radial Galbraith plot did not provide suspicion of bias in a particular direction due to different precisions. No sources of external bias were identified (Table 2).

**References**

1. Marcus R, Peritz E, Gabriel KR: **On Closed Testing Procedures with Special Reference to Ordered Analysis of Variance.** *Biometrika* 1976, **63:**655–660.

2. Higgins JPT, Green S (Eds.). **Cochrane handbook for systematic reviews of interventions version 5.1.0.** *The Cochrane Collaboration* 2011**.** [www.cochrane-handbook.org]
