# Supplementary material for: Recombinant human follicle-stimulating hormone (r-hFSH) plus recombinant luteinizing hormone versus r-hFSH alone for ovarian stimulation during assisted reproductive technology: systematic review and meta-analysis
Source: Reprod Biol Endocrinol. 2014 Feb 20;12:17. doi: 10.1186/1477-7827-12-17 (PMC4015269; doi:10.1186/1477-7827-12-17)
Supplement: Additional file 2: Table S1 — Search strategy for the MEDLINE database. [file 1477-7827-12-17-S2.doc]

**Supplementary Table S1 Search strategy for the MEDLINE database**

| **Language** |
| --- |
| Unrestricted |
| **Date** |
| January 1, 1990, to May 1, 2011 |
| **Key terms and expressions** |
| 1. (luveris or lhadi or reclh or rlh or rhlh).mp  2. ((alpha or alfa or recombinant or rec or r or rh or r-h) adj2 (lutropin or luteoz?man)).tw  3. (recombinant adj3 ((luteini?ing adj hormone$) or lh or hlh or lhs or hlhs)).tw  4. (rec adj2 ((luteini?ing adj hormone$) or lh or hlh or lhs or hlhs)).tw  5. (r adj2 ((luteini?ing adj hormone$) or hlh or lhs or hlhs)).tw  6. (r adj lh).mp  7. ((recombinant adj2 gonadotropin$) and ((luteini?ing adj hormone$) or lh or hlh or lhs or hlhs)).tw  8. ((exogenous or combination or (co adj (administrat$ or treatment))) adj2 (lutropin or luteoz?man or (luteini?ing adj hormone$) or lh or hlh or lhs or hlhs)).tw  9. (added adj (lutropin or luteoz?man or (luteini?ing adj hormone$) or lh or hlh or lhs or hlhs)).tw  10. exp Recombinant Proteins/ and exp Luteinizing Hormone/  11. exp Luteinizing Hormone/ad, tu  12. FSH.mp. or exp follicle stimulating hormone/  13. 11 and 12  14. (or/1-10) or  15. (clinical trial.mp. or randomi?ed.ti,ab, or placebo.ti,ab. Or exp clinical trials/ or randomly.ti,ab or trial.ti,ab) not (animals/ not (animals/ and humans/))  16. 14 and 15 |
