# Supplementary material for: Recombinant human follicle-stimulating hormone (r-hFSH) plus recombinant luteinizing hormone versus r-hFSH alone for ovarian stimulation during assisted reproductive technology: systematic review and meta-analysis
Source: Reprod Biol Endocrinol. 2014 Feb 20;12:17. doi: 10.1186/1477-7827-12-17 (PMC4015269; doi:10.1186/1477-7827-12-17)
Supplement: Additional file 3: Table S2 — Missing data imputation calculations and coefficient determinations. [file 1477-7827-12-17-S3.doc]

**Supplementary Table 2 Missing data imputation calculations and coefficient determinations**

| **Variable** | ***R*2** | **95% CI** | **Transformation** | **Slope** |
| --- | --- | --- | --- | --- |
| NOM=f(NOV) | 0.81 | 0.68–0.89 | Log | 0.876 |
| NE=f(NOM) | 0.79 | 0.32–0.95 | Log | 0.732 |
| NQE=f(NE) | 0.81 | 0.36–0.95 | Log | 0.608 |
| BPR=f(CPR) | 0.98 | 0.96–0.99 | – | 0.758 |
| OPR=f(CPR) | 0.98 | 0.96–0.99 | – | 0.827 |
| LBR=f(CPR) | 0.96 | 0.92–0.98 | – | 0.764 |

BPR, biochemical pregnancy rate; CI, confidence interval; CPR, clinical pregnancy rate; f, function; LBR, live birth rate; NE, number of embryos; NOM, number of metaphase II oocytes; NOV, number of oocytes; NQE, number of good-quality embryos; OPR, ongoing pregnancy rate; *R*2, coefficient determination.
