# Supplementary figures and images for: Recombinant human follicle-stimulating hormone (r-hFSH) plus recombinant luteinizing hormone versus r-hFSH alone for ovarian stimulation during assisted reproductive technology: systematic review and meta-analysis
Source: Reprod Biol Endocrinol. 2014 Feb 20;12:17. doi: 10.1186/1477-7827-12-17 (PMC4015269; doi:10.1186/1477-7827-12-17)

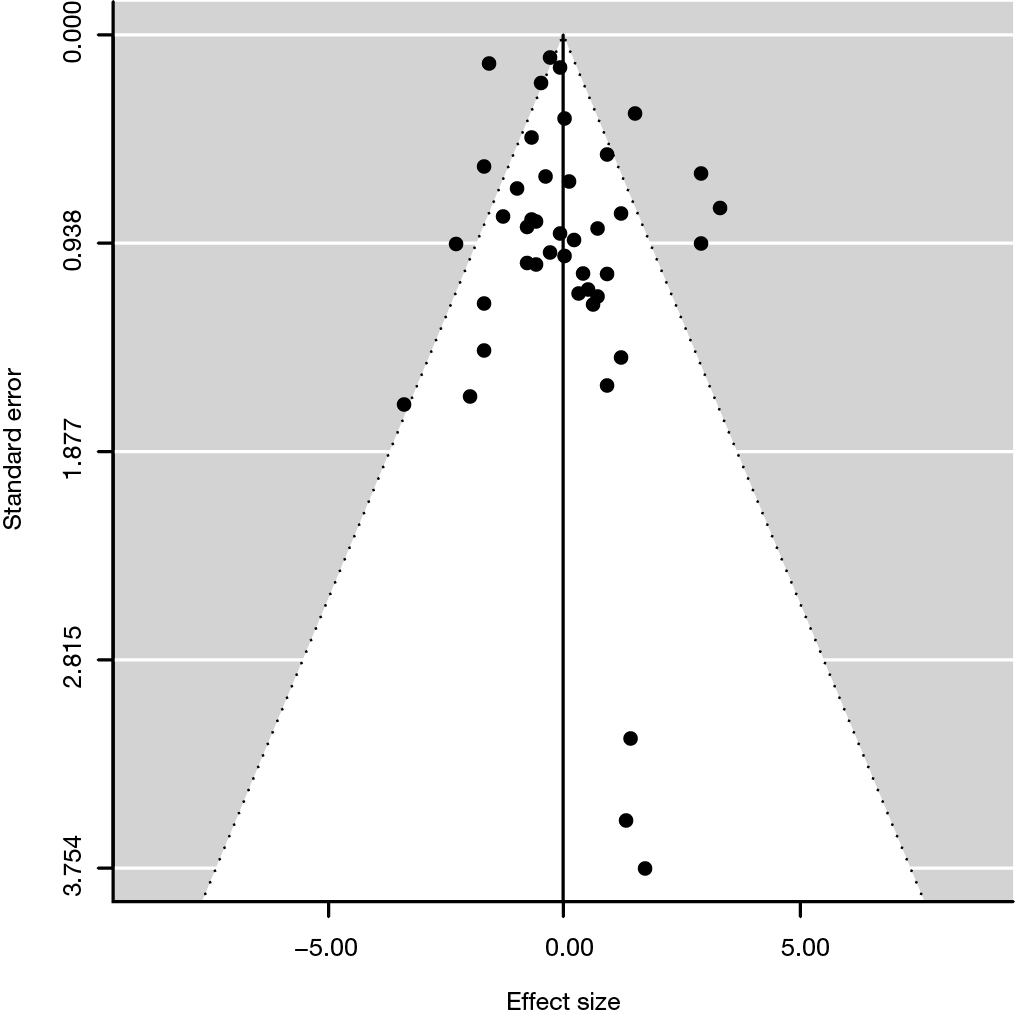

Supplement: Additional file 4: Figure S1 — Funnel plot of effect size by standard error for number of oocytes. [file 1477-7827-12-17-S4.jpeg]

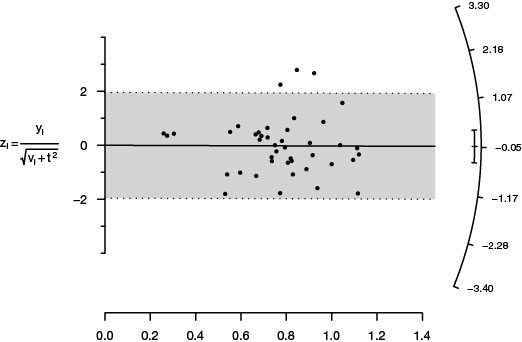

Supplement: Additional file 5: Figure S2 — Radial Galbraith plot for number of oocytes to assess the consistency of the observed outcomes with different precisions. [file 1477-7827-12-17-S5.jpeg]
