# Supplementary material for: Recombinant human follicle-stimulating hormone (r-hFSH) plus recombinant luteinizing hormone versus r-hFSH alone for ovarian stimulation during assisted reproductive technology: systematic review and meta-analysis
Source: Reprod Biol Endocrinol. 2014 Feb 20;12:17. doi: 10.1186/1477-7827-12-17 (PMC4015269; doi:10.1186/1477-7827-12-17)
Supplement: Additional file 6: Table S3 — Study typology analysis for the co-primary endpoints (difference between the r-hFSH plus r-hLH and r-hFSH alone groups). [file 1477-7827-12-17-S6.doc]

**Supplementary Table 3 Study typology analysis for the co-primary endpoints (difference between the r-hFSH plus r-hLH and r-hFSH alone groups)**

| **Patient subgroup** | **Number of oocytes retrieved** | |  | **Clinical pregnancy rate** | |
| --- | --- | --- | --- | --- | --- |
|  | **Mean difference (95% CI)** | ***P* value** |  | **Relative risk (95% CI)** | ***P* value** |
| NNG | −0.36 (−0.98 to 0.26) | 0.25 |  | 0.07 (−0.05 to 0.18) | 0.24 |
| NPG | 1.40 (0.35 to 2.46) | 0.01 |  | 0.27 (−0.02 to 0.56) | 0.07 |
| ANG | −0.26 (−1.45 to 0.94) | 0.67 |  | 0.07 (−0.21 to 0.34) | 0.63 |
| NNN | 0.36 (−0.89 to 1.61) | 0.58 |  | −0.14 (−0.34 to 0.07) | 0.19 |
| NPN | 0.77 (−0.86 to 2.39) | 0.35 |  | 0.19 (−0.38 to 0.76) | 0.51 |
| APG | 0.61 (−0.96 to 2.18) | 0.45 |  | −0.04 (−0.52 to 0.45) | 0.88 |
| ANN | −1.34 (−3.57 to 0.89) | 0.24 |  | 0.16 (−0.13 to 0.45) | 0.29 |

Subgroups: ANG, advanced maternal age, normal response, GnRH agonist; ANN, advanced maternal age, normal response, GnRH antagonist; APG, advanced maternal age, poor response, GnRH agonist; NNG, young/normal age, normal response, GnRH agonist; NNN, young/normal age, normal response, GnRH antagonist; NPG, young/normal age, poor response, GnRH agonist; NPN, young/normal age, poor response, GnRH antagonist.

CI, confidence interval; GnRH, gonadotrophin-releasing hormone; r-hFSH, recombinant human follicle-stimulating hormone; r-hLH, recombinant human luteinizing hormone.
